# Translation and Validation of the Korean Version of the Postoperative Quality of Recovery Score QoR-15

**DOI:** 10.1155/2020/3456234

**Published:** 2020-10-09

**Authors:** Donggyeong Kim, Jay Kyoung Kim, Jinseok Yeo

**Affiliations:** ^1^Department of Anesthesiology and Pain Medicine, The Armed Forces Daegu Hospital, Gyeongsangbuk-do, Republic of Korea; ^2^Department of Anesthesiology and Pain Medicine, Kyungpook National University Chilgok Hospital, School of Medicine, Kyungpook National University, Daegu, Republic of Korea

## Abstract

Quality of recovery after anesthesia is an important measure of the early postoperative health status of patients. The Quality of Recovery-15 (QoR-15) questionnaire is a self-rated questionnaire used to assess the quality of postoperative recovery. This study is aimed at translating and validating the Korean version of QoR-15 (QoR-15K). One hundred fifty patients were included in this study. We translated the original version of QoR-15 into QoR-15K and evaluated its validity, reliability, responsiveness, and clinical feasibility. QoR-15K showed acceptable criterion, structure, and construct validities. Reliability was verified using Cronbach's *α* (0.856), standard error of measurement (10.78), split-half reliability (0.831), test-retest reliability (*ρ* = 0.945, *P* ≤ 0.001), and intraclass correlation test-retest (*ρ* = 0.903, *P* ≤ 0.001). These results represent an acceptable reliability. Responsiveness was verified using Cohen's effect size (1.39), standardized response mean (1.03), and the correlation between QoR-15K score and duration of anesthesia (*ρ* = −0.197, *P* = 0.016). These results show acceptable responsiveness. The mean ± standard deviation time to complete QoR-15K was 138.1 ± 30.7 s. QoR-15K was rated more than adequate on the COnsensus-based Standards for the selection of health Measurement INstruments checklist. In conclusion, QoR-15K shows acceptable validity, reliability, responsiveness, and clinical feasibility and may help evaluate postoperative quality of recovery in Korean populations.

## 1. Introduction

Recovery after surgery is a complex process. Many factors affect the recovery process, including the patient's medical condition, type of surgery, adverse sequelae after surgery, and anesthesia. With the introduction of minimally invasive surgery and enhanced recovery after surgery, there is an increased need for an assessment tool to evaluate the quality of recovery [1]. The quality of recovery is assessed as recovery time, pain, nausea, postoperative complication rates, and adverse events. Although these parameters are essential objective components for the quality of recovery, they do not fully reflect the patient's social and psychological recovery experience. Different patient-centered measurement tools have been developed and validated to evaluate the patient's recovery experience [2–5].

The Quality of Recovery-15 (QoR-15) questionnaire is a short 15-item scale derived from QoR-40 [3]. It assesses five domains of a patient's health: emotional state, physical comfort, psychological support, physical independence, and pain. Each item uses an 11-point numeric rating scale. The sum of the scores of the 15 items ranges from 0 to 150, with a high score indicating good quality of recovery. The questionnaire decreases the evaluation time, improves convenience, and has strong evidence for good content validity and internal consistency. However, a Korean version of QoR-15 (QoR-15K) has not been established. The development of QoR-15K and its validation would improve the quality of recovery after surgery in the Korean population. Therefore, we developed and validated QoR-15K according to the COnsensus-based Standards for the selection of health Measurement INstruments (COSMIN) taxonomy in surgical patients [6].

## 2. Patients and Methods

### 2.1. Translation and Cultural Adaption of QoR-15

After obtaining consent from the original author, two independent translators (native speakers of the Korean language) translated the English version of QoR-15 into Korean. We first made a temporary Korean version of QoR-15, and then, two different translators (native speakers of the English language) back-translated this version into English. Therefore, each question was rendered in its most intelligible form by a committee of four translators. The committee modified “feeling rested” to “feeling rested enough,” “doctors and nurses” to “medical personnel,” and “feeling comfortable and in control” to “feeling comfortable and going well” in Korean. The committee concluded that these changes made it easier to understand the questionnaire in Korean while conveying the nuances of the English version. After the committee came to a consensus on the Korean version of QoR-15, it was tested on 10 randomly selected postoperative patients. The test revealed that all questions were understandable. As a result, the Korean version of QoR-15 (QoR-15K) was finalized.

### 2.2. Patients

After the institutional review board approved this research, a prospective observational study was conducted. Patients provided oral consent. Patients with poor Korean comprehension or those with cognitive impairment were excluded from the study. Furthermore, those with a known history of substance abuse, any severe preexisting medical conditions that limited objective assessment after the operation, or any life-threatening postoperative complications were excluded.

Demographic and perioperative data were collected via the hospital's medical information system. We recorded sex, age, American Society of Anesthesiologists physical status score (ASA PS), duration of surgery and anesthesia, extent of surgery, duration of postoperative stay, and postoperative complications within 24 h. We classified the extent of surgery as minor, intermediate, or major. Laparoscopic abdominal surgery with minimal incision lengths and surgery on the surface of the body was classified as minor surgery, laparoscopic abdominal surgery using a hand port as intermediate surgery, and open abdominal surgery as major surgery.

### 2.3. Protocol

Before the day of surgery, investigators asked the patients to complete the QoR-15K questionnaire as a measure of baseline status. The patients were then asked to repeat the questionnaire at 24 h postoperatively. Patients were also asked to rate their overall postoperative recovery using an 11-point numerical rating scale (NRS), which ranged from “poor recovery (0)” to “excellent recovery (10).” All patients were asked to repeat the QoR-15K questionnaire within 30–60 min. We selected 50 patients from those who completed the QoR-15K questionnaire. The selected patients were asked to repeat the QoR-15K and SF-36 Korean version 2.0 questionnaires on postoperative day 7 to verify the correlation between the two questionnaires.

### 2.4. Psychometric Evaluation

Domain reliability represents the degree to which the measurement is free from measurement errors. It contains internal consistency, reliability, and measurement error. To assess internal consistency and measurement error, we used Cronbach's *α* and split-half reliability. To assess reliability, we used test-retest reliability at 24 h postoperatively. Measurement error was estimated according to COSMIN taxonomy [6].

Domain validity denotes the degree to which an outcome measures the construct of interest. It comprises content, criterion, and construct validities. Content validity is the degree to which an instrument is an adequate reflection of the construct to be measured. It was evaluated via interviews with patients and researchers. To assess criterion validity, we tested predictive validity by calculating the correlation between postoperative hospitalization duration and QoR-15K scores at 24 h postoperatively. Construct validity comprises structural validity, hypothesis testing, and cross-cultural validity. Structural validity assesses whether the instrument is an adequate reflection of the dimensionality of the construct to be measured and is determined via exploratory factor analysis. Hypothesis testing examines how much the instrument measure relates to other measures in a way that is expected if the instrument is validly measuring the supposed construct. We hypothesized that the patient who had experienced poor postoperative recovery would have a lower QoR-15K score. We assessed the difference of mean score for QoR-15K according to the patients who had good or poor recovery. An NRS score of recovery ≥ 7 represented good recovery, while an NRS score of recovery < 7 represented poor recovery [3]. Cross-cultural validity describes the degree to which a translated or culturally adapted questionnaire is an adequate reflection of the original questionnaire. Because we only translated and changed nuances, we only evaluated the translation process.

Responsiveness is an instrument's ability to measure change over time. Responsiveness was measured via standardized response mean (SRM), which was calculated by dividing the mean change in the score by the standard deviation (SD) of the difference [7]. Cohen's effect size was calculated as the average change in the QoR-15K score (from preoperative to postoperative) divided by the pooled SD [8]. We also performed hypothesis testing to assess responsiveness according to COSMIN taxonomy [6]. We hypothesized a negative correlation between surgery duration and QoR-15K.

Recruitment rate, rate of completion, and time taken to complete QoR-15K were used to test clinical feasibility.

### 2.5. Assessment of Methodological Quality

The COSMIN four-point checklist, consisting of 10 boxes, was used to assess the methodological quality of the study [9]. Each item in a box is rated as a four-point checklist, and the measurement is determined using the lowest rating of any items in a box. We did not evaluate the patient-reported outcome measure development box because we did not develop QoR-15K.

### 2.6. Statistical Analysis

We selected a sample size of 150 patients with reference to previous research in the same field and based on the advice of a university statistician [3, 4]. Demographic and perioperative data were presented as means (SD). Qualitative data were presented as frequency and percentage. The Kruskal-Wallis test was used to analyze differences in distribution. Comparisons of the QoR-15K scores were analyzed using the Mann-Whitney *U* test. Associations were measured using Pearson's correlation coefficient (*r*) and Spearman's correlation coefficient (*ρ*). All analyses were performed using SPSS Statistics for Windows, version 21.0 (SPSS; IBM Corp, Armonk, NY). The statistical significance for all analyses was set at *P* < 0.05.

## 3. Results

### 3.1. Patients and Questionnaire Data

Of the 165 patients who were asked to participate in this study, 8 were not eligible, and 2 refused to participate. Five patients were excluded after recruitment due to incomplete questionnaires. Finally, a total of 150 patients completed their pre- and postoperative questionnaires. All patients received general anesthesia. The demographic and clinical characteristics of the 150 patients are given in Table 1.

The completion rate of QoR-15K was 100% preoperatively and 97% at 24 h postoperatively. The mean duration of hospitalization was 8.4 ± 6.2 days. It took 157.0 ± 33.0 s to complete QoR-15K preoperatively and 138.1 ± 30.7 s at 24 h postoperatively. Most patients were able to complete QoR-15K without assistance. These results show that QoR-15K is acceptable and clinically feasible. The baseline and postoperative QoR-15 scores were 118.3 ± 24.4 and 84.4 ± 28.4, respectively, with a significant difference (*P* ≤ 0.001). Postoperative QoR-15K scores were in the range of 18–150. One patient reported a postoperative QoR-15K score of 150. There was a positive correlation between age and time taken to complete the questionnaire (*ρ* = 0.332; *P* ≤ 0.001) and a negative correlation between the total QoR-15K score and time taken to complete QoR-15K (*ρ* = −0.276; *P* ≤ 0.001).

### 3.2. Psychometric Properties

In the domain “Reliability,” Cronbach's *α*, standard error of measurement, and split-half reliability were 0.856, 10.78, and 0.831, respectively. The test-retest reliability (*ρ*) was 0.945 (95% confidence interval [CI]: 0.919–0.962; *P* ≤ 0.001), and the interclass correlation coefficient test-retest (*ρ*) was 0.903 (95% CI: 0.869–0.929; *P* ≤ 0.001).

In the domain “Validity,” content validity, according to the COSMIN four-point checklist, was determined to be adequate (Table 2). We use predictive validity to assess criterion validity. The postoperative QoR-15K score negatively correlated with postoperative length of hospital stay (*ρ* = 0.265; *P* = 0.004). These results show that a lower QoR-15K score predicts a longer postoperative stay; therefore, QoR-15K has predictive validity. To assess structural validity, we performed exploratory factor analysis, showing acceptable results (Kaiser − Meyer − Olkin measure = 0.821; *P* = 0.000). To assess construct validity, we performed hypothesis testing as follows. We hypothesized that the patient who had experienced poor postoperative recovery would have a lower QoR-15K score. The QoR-15K score significantly differed between the good (*n* = 82) and poor (*n* = 68) recovery groups (96.2 ± 25.3 and 70.2 ± 25.3 (*P* ≤ 0.001), respectively). The NRS of recovery showed a positive correlation with postoperative QoR-15K (*ρ* = 0.579; *P* ≤ 0.001), demonstrating construct validity. Cross-cultural validity, according to the COSMIN four-point checklist, was determined to be adequate.

In the domain “Responsiveness,” we assessed responsiveness and hypothesis testing. Cohen's effect size was 1.28, and SRM was 1.03 (Table 3). These results showed that QoR-15K has a large effect size and high responsiveness. We hypothesized that the patient with longer surgery and anesthesia time would have a lower QoR-15K score. The postoperative QoR-15K score negatively correlated with duration of surgery (*ρ* = −0.212; *P* = 0.009) and anesthesia (*ρ* = −0.197; *P* = 0.016). The methodological quality of the study, according to the COSMIN checklist, was rated more than adequate.

We also compared the QoR-15K score with the level of surgery (minor, intermediate, and major). There were significant differences in the distribution of sex, age, ASA physical status score, duration of surgery and anesthesia, and postoperative QoR-15K scores in patients in the minor surgery group (Table 4). The minor surgery group included breast, thyroid, and laparoscopic gynecologic surgeries. Therefore, this group is primarily comprised of women and younger adults. The postoperative QoR-15K scores revealed differences between the three groups (*P* = 0.004). The minor surgery group showed a significantly higher score than the intermediate surgery (*P* = 0.004) and major surgery (*P* = 0.015) groups after post hoc testing using the Tukey HSD test. Twenty-one patients had postoperative complications, including headache, dizziness, fever, and nausea. There were no life-threatening postoperative complications. Postoperative QoR-15K scores between patients with and without postoperative complications did not show a significant difference. According to sex and ASA physical status, there were no significant differences in postoperative QoR-15K scores. In addition, there was also no relationship between the postoperative QoR-15K score and patient's age.

The items within QOR-15 can be divided into two components: physical and mental well-being [3]. The QoR-15K score strongly correlated with the sum of the items in these two components (physical well-being; *ρ* = 0.913; *P* ≤ 0.001 and mental well-being; *ρ* = 0.857; *P* ≤ 0.001) (Table 5). The QoR-15K score also strongly correlated with the physical component scale (*ρ* = 0.663; *P* ≤ 0.001) and moderately with the mental component scale (*ρ* = 0.44; *P* ≤ 0.001) of short-form (SF-36) health survey on postoperative day 7. The sum of physical well-being items on QoR-15K strongly correlated with the physical component scale of SF-36 (*ρ* = 0.642; *P* ≤ 0.001) (Table 6).

## 4. Discussion

In the current study, we assessed QoR-15K in patients recovering from general anesthesia. The results of our study indicate that QoR-15K has acceptable levels of validity, reliability, responsiveness, and clinical feasibility in the Korean population.

Several findings indicate that QoR-15K has acceptable validity. First, the postoperative QoR-15K score showed a negative correlation with the postoperative length of hospital stay. Second, the QoR-15K score showed a significant correlation with recovery NRS. The QoR-15K scores in the good recovery group were significantly higher than those in the poor recovery group. These results are consistent with the original study and support construct validity. Third, the fact that the baseline and postoperative QoR-15K scores were significantly different in this study could be a good indicator of construct validity.

QoR-15K demonstrated excellent internal consistency with Cronbach's *α* and split-half reliability [10, 11] exceeding the recommended value (Cronbach′s *α* = 0.7 and split − half reliability = 0.75) [12]. These results confirm the acceptable reliability of QoR-15K. Corrected item-total correlation (0.23–0.72) indicates that the items in QoR-15K are appropriate and relevant with no unnecessary items. The corrected item-total correlation of item 13 (0.23), indicating nausea or vomiting, was relatively low in comparison with the original study (0.47). This finding may be related to the relatively low incidence rate of nausea or vomiting in this study. Test-retest reliability (*ρ* = 0.95) was excellent and similar to that in the original study (*ρ* = 0.99). All items in QoR-15K performed at above acceptable levels (*ρ* = 0.80–0.91), which is consistent with the original study [3].

Consistent with previous findings, QoR-15K was not related to demographic factors, such as sex, age, and ASA physical status, in this study [3, 13]. This result shows that QoR-15 is less affected by these demographic variables. However, QoR-40 differs between sexes, with a higher score for males. This difference indicates that QoR-15 may not reflect the general notion that women typically experience a lower quality of recovery [4].

Responsiveness is also acceptable. The SRM value of the total of the QoR-15K scores was 1.03, demonstrating the ability to assess alterations in the postoperative recovery period [14]. However, the SRM value of the items concerning emotional status (feeling worried or anxious or feeling sad or depressed) assessed in this study was low compared with that in the original research (0.10 vs. 0.35 and 0.06 vs. 0.20, respectively) [3]. A Japanese study also showed lower SRM in emotional status [15]. East Asian people usually express less emotion in response to especially sad situations [16]. We believe this cultural characteristic may have affected our results. QoR-15K had a negative correlation with the length of postoperative hospital stay (*ρ* = –0.28) and duration of surgery and anesthesia (*ρ* = –0.21 for both). These associations were similar to the Portuguese version (*ρ* = –0.28 for the length of hospital stay) yet modestly weaker than those in the original evaluation of QoR-15 (*ρ* = –0.53 for the length of hospital stay, *ρ* = –0.49 for the duration of surgery).

Most patients were able to complete the QoR-15K questionnaire in 3 min. This result is similar to that of the English and Portuguese versions [3, 13]. As expected, older participants and patients with lower QoR-15K scores took more time to complete the questionnaire. It is also notable that 97% of participants completed QoR-15K within 24 h postoperatively. These data indicate good clinical feasibility.

This study has certain limitations. First, we did not include patients who underwent ambulatory or orthopedic surgeries and a patient who received regional anesthesia. Patients who underwent neurosurgical and cardiac surgeries were also not included in this study. However, our study included patients with all levels of surgery, and we do not believe that these exclusions affected our results. Second, postoperative complications within 24 h of surgery did not affect the QoR-15K score. We included patients who underwent elective surgery, and there were only a small number of reported complications in this study, which was minor. We believe these results could change if we include patients who undergo emergency surgery and a more significant number of patients.

## 5. Conclusion

In conclusion, we translated QoR-15 into Korean and validated it according to the COSMIN taxonomy. QoR-15K has acceptable validity, reliability, responsiveness, and clinical feasibility and may help in measuring the quality of postoperative recovery in the Korean population undergoing surgery.

## Figures and Tables

**Table 1:** Demographic data of the patients.

Characteristics	Value
Age (years)	58.5 ± 12.8 (26–88) Male 61.6 ± 11.7 and female 55.5 ± 13.0
Sex (male/female)	74/76
ASA PS (I/II/III)	60/83/7
Type of surgery	
Breast surgery	4
Thyroid surgery	4
Colorectal surgery	72
Hepatobiliary surgery	4
Stomach surgery	22
Gynecologic surgery	27
Urologic surgery	17
Duration of surgery (min)	169.4 ± 88.7 (25–485)
Duration of anesthesia (min)	210.1 ± 93.9 (44–545)
Length of postoperative hospital stay (days)	8.4 ± 6.2 (2–49)
Postoperative complications within 24 h of surgery	21
Preoperative QoR-15K score	118.3 ± 24.4 (86–150)
Postoperative QoR-15K score	84.4 ± 28.4 (18–150)

ASA PS: American Society of Anesthesiologists performance status; QoR-15K: Korean version of QoR-15. Data are presented as mean ± standard deviation (range) or frequencies.

**Table 2:** Methodological quality ratings based on the COSMIN checklist with four-point scale.

Box	Rating
Content validity	Adequate
Structural validity	Adequate
Internal consistency	Adequate
Cross-cultural validity	Adequate
Reliability	Adequate
Measurement error	Adequate
Criterion validity	Very good
Hypothesis testing for construct validity	Very good
Responsiveness	Adequate

**Table 3:** Mean, change, responsiveness, test-retest reliability, and corrected total item correlation of postoperative QoR-15K.

QoR-15K item	Postoperative score	Baseline score	Change from baseline	Cohen's effect size	Standardized response mean	Test-retest reliability	Corrected item total correlation
(1) Able to breathe easy	7.3 ± 2.8	8.8 ± 2.7	−1.5 ± 3.8	0.55	0.40	0.862 (0.809–0.900)	0.524
(2) Been able to enjoy food	2.1 ± 3.6	7.7 ± 3.5	−5.6 ± 4.6	1.58	1.22	0.832 (0.768–0.878)	0.428
(3) Feeling rested	4.9 ± 3.4	7.6 ± 2.8	−2.4 ± 4.0	0.86	0.60	0.886 (0.835–0.920)	0.720
(4) Have had a good sleep	5.1 ± 3.6	7.5 ± 2.9	−2.3 ± 4.2	0.70	0.55	0.894 (0.852–0.923)	0.518
(5) Able to look after personal toilet and hygiene unaided	4.3 ± 4.1	9.3 ± 2.3	−5.0 ± 4.6	1.50	1.08	0.908 (0.873–0.934)	0.486
(6) Able to communicate with family or friends	8.1 ± 2.8	9.3 ± 2.2	−1.2 ± 3.4	0.48	0.35	0.873 (0.825–0.908)	0.567
(7) Getting support from hospital doctors and nurses	7.4 ± 3.4	5.8 ± 4.4	1.6 ± 4.9	0.41	0.33	0.799 (0.722–0.854)	0.315
(8) Able to return to work or usual home activities	2.2 ± 3.4	8.2 ± 2.9	−6.0 ± 4.0	1.90	1.50	0.903 (0.867–0.930)	0.513
(9) Feeling comfortable and in control	7.1 ± 2.9	7.9 ± 2.8	−0.7 ± 3.5	0.25	0.20	0.893 (0.853–0.923)	0.618
(10) Having a feeling of general well-being	6.1 ± 3.1	7.7 ± 2.8	−1.6 ± 3.7	0.54	0.43	0.903 (0.866–0.930)	0.626
(11) Moderate pain	4.4 ± 2.9	8.0 ± 2.9	−3.7 ± 4.0	1.27	0.93	0.781 (0.697–0.841)	0.518
(12) Severe pain	5.0 ± 3.3	8.5 ± 2.8	−3.6 ± 4.1	1.18	0.88	0.801 (0.725–0.856)	0.592
(13) Nausea or vomiting	7.8 ± 3.2	8.8 ± 2.7	−1.0 ± 3.6	0.34	0.28	0.816 (0.746–0.867)	0.225
(14) Feeling worried or anxious	5.9 ± 3.2	6.3 ± 3.2	−0.4 ± 3.8	0.13	0.11	0.870 (0.820–0.906)	0.534
(15) Feeling sad or depressed	6.8 ± 3.3	7.00 ± 3.1	−0.2 ± 3.5	0.06	0.06	0.905 (0.868–0.931)	0.513
Total	84.4 ± 28.4	118.3 ± 24.4	−33.9 ± 33.0	1.28	1.03	0.945 (0.919–0.962)	NA

QoR-15K: Korean version of QoR-15; SRM: standard response mean. Data are presented as mean ± standard deviation. Test-retest was presented as Spearman's *ρ* correlation coefficient and 95% confidence interval.

**Table 4:** Patient characteristics according to the extent of surgery.

Variable	Minor (*n* = 33)	Intermediate (*n* = 75)	Major (*n* = 42)	*P* value
Age	46.6 ± 9.9	62.9 ± 11.4	60.0 ± 11.2	≤0.001
Sex (male/female)	2/31	45/30	27/15	≤0.001
ASA PS class (I/II/III)	21/12/0	24/46/5	15/25/2	0.03
Duration of surgery	112.2 ± 61.3	163.3 ± 85.3	225.2 ± 81.4	≤0.001
Duration of anesthesia	144.9 ± 69.8	208.0 ± 89.5	264.9 ± 85.3	≤0.001
Postoperative hospital stay (days)	5.0 ± 4.2	8.9 ± 5.0	10.1 ± 8.1	≤0.001
Postoperative complications within 24 h of surgery	3	13	5	0.28
Preoperative QoR-15K	124.2 ± 2	115.4 ± 25.0	118.6 ± 26.5	0.22
Postoperative QoR-15K	98.7 ± 29.2	80.2 ± 25.4	80.7 ± 29.7	≤0.001

ASA PS: American Society of Anesthesiologists performance status; QoR-15K: Korean version of QoR-15. Minor: laparoscopic abdominal surgery with minimal incision lengths and surgery on the surface of the body; intermediate: laparoscopic abdominal surgery using a hand port; major: open abdominal surgery. Data are presented as mean ± standard deviation (range) or frequencies.

**Table 5:** Interdimensional correlation of QoR-15K.

	Total QoR-15K	Physical well-being	Mental well-being
Physical well-being	*ρ* = 0.913; *P* ≤ 0.001		
Mental well-being	*ρ* = 0.857; *P* ≤ 0.001	*ρ* = 0.653; *P* ≤ 0.001	

QoR-15K: Korean version of QoR-15. Physical well-being: sum of items of 1, 2, 3, 4, 5, 8, 11, 12, and 13 in QoR-15K; mental well-being: sum of items of 6, 7, 9, 10, 14, and 15 in QoR-15K.

**Table 6:** Correlation between QoR-15K and SF-36 scores.

	SF-36 scores	
	Physical component score	Mental component score
Total QoR-15K	*ρ* = 0.663; *P* ≤ 0.001	*ρ* = 0.444; *P* = 0.001
Mental well-being	*ρ* = 0.583; *P* ≤ 0.001	*ρ* = 0.443; *P* = 0.001
Physical well-being	*ρ* = 0.642; *P* ≤ 0.001	*ρ* = 0.427; *P* = 0.002

QoR-15K: Korean version of QoR-15; SF-36: short-form health survey Korean version 2.0. Mental well-being: sum of items of 6, 7, 9, 10, 14, and 15 in QoR-15K; physical well-being: sum of items of 1, 2, 3, 4, 5, 8, 11, 12, and 13 in QoR-15K.

## Data Availability

The datasets generated during and/or analyzed during the current study are available from the corresponding author on reasonable request.
